# Tongxie Anchang Decoction Relieves Visceral Hypersensitivity in Diarrhea-Predominant Irritable Bowel Syndrome Rats by Regulating the NGF/TrkA Signaling Pathway

**DOI:** 10.1155/2021/6679348

**Published:** 2021-06-18

**Authors:** Xiang Tan, Wenjing Pei, Chune Xie, Zhibin Wang, Jiali Liu, Yuan Cheng, Tangyou Mao, Lei Shi, Xingjie Zhao, Qiongqiong Lu, Zhongmei Sun, Fushun Kou, Hui Jiang, Junxiang Li

**Affiliations:** ^1^Graduate School of Beijing University of Chinese Medicine, Beijing 100029, China; ^2^Department of Gastroenterology, Dongfang Hospital, Beijing University of Chinese Medicine, Beijing 100078, China; ^3^Xiyuan Hospital, China Academy of Chinese Medical Sciences, Beijing 100091, China; ^4^School of Life Sciences, Beijing University of Chinese Medicine, Beijing 100029, China

## Abstract

Irritable bowel syndrome (IBS) is a functional gastrointestinal disease characterized by visceral hypersensitivity-related abdominal pain, in which diarrhea-predominant IBS (IBS-D) is the main subtype and has a high clinical incidence. Tongxie Anchang Decoction (TXACD) has been proved to significantly improve abdominal pain in patients with IBS-D, but its underlying therapeutic mechanism still remains unclear. In the present study, IBS-D model rats were induced by neonatal maternal separation (NMS) combined with restraint stress (RS). The therapeutic effect of TXACD was evaluated by fecal characteristics and abdominal withdrawal reflex (AWR) scores. After 14 days of intragastric administration, the colonic tissues of rats were collected to detect the protein and gene level of the NGF, TrkA, and TRPV1 using Western blotting and real-time polymerase chain reaction, respectively, and detect mast cells infiltration using toluidine blue staining. The abdominal aorta blood centrifuged was collected for detecting serum levels of SP, 5-HT, and CGRP with ELISA. The results revealed that TXACD could significantly improve visceral hypersensitivity in IBS-D rats, reflected in the decrease of AWR score and the serum levels of SP, 5-HT, and CGRP. In addition, TXACD treatment could alleviate mast cells infiltration. Moreover, the expression levels of the NGF, TrkA, and TRPV1 were repressed by TXACD. The findings of the present study indicated that the therapeutic effect of TXACD on visceral hypersensitivity might be closely related to the downregulation of the NGF/TrkA signaling pathway, the reversal of TRPV1 expression and mast cells infiltration, and the decreased release of neuroendocrine factors SP, 5-HT, and CGRP.

## 1. Introduction

Irritable bowel syndrome (IBS) is the most common functional gastrointestinal disease characterized by visceral hypersensitivity-related abdominal pain accompanied by changes in defecation habits and characteristics [1, 2]. According to Roman IV, IBS can be divided into four subtypes: IBS with diarrhea (IBS-D), IBS with constipation (IBS-C), IBS with mixed bowel habit (IBS-M), and IBS unclassified (IBS-U), of which IBS-D is the most common in clinic [3]. The global prevalence of IBS is 5–20% [4–6], and the prevalence in China is about 1–16% [7]. IBS seriously affects the work and life of patients, causing a huge economic burden [8]. According to conservative estimates, the direct economic loss caused by IBS amounts to US $1 billion [9].

The etiology and pathogenesis of IBS-D are not very clear. A variety of pathological factors are involved in the occurrence and development of IBS-D, among which visceral hypersensitivity is considered to be very important [10]. Abnormal brain-gut axis regulation is closely associated with the formation of visceral hypersensitivity. Studies have shown that long-term exposure to adverse stimuli, such as poverty and lack of parental care, can adversely affect health and increase the risk of functional gastrointestinal diseases (such as IBS) later on life [11]. The hypothalamic-pituitary-adrenal (HPA) axis plays a key role in regulating the homeostatic response to stress. The interaction between the HPA axis and nerve growth factor (NGF) plays an important part in the occurrence of early life stress-induced functional gastrointestinal disorders [12]. Acute or chronic stress increases the expression of the corticotrophin-releasing factor (CRF) in the central nervous system and intestinal tissue, which in turn increases the expression of the NGF in intestinal mucosa and promotes the release of the NGF in intestinal mast cells. On the other hand, the increased NGF in the intestine also affects the homeostasis of HPA axis [13, 14].

The NGF exerts its biological function through promyosin kinase receptor A (TrkA). The NGF/TrkA signaling pathway is closely related to visceral hypersensitivity. Studies have shown that the expression of the NGF and its cognate receptor TrkA is significantly increased in the spinal cord and colon of rats treated with neonatal maternal separation (NMS). And the use of the recombinant NGF in neonatal rats can lead to visceral hypersensitivity and mucosal barrier destruction. It is worth noting that the inhibition of NGF signal by using NGF antagonists effectively alleviates the intestinal dysfunction induced by NMS [15].

At present, symptomatic treatment is often adopted for IBS-D in clinic, but the curative effect is uncertain and the symptoms of IBS-D are easy to relapse. Because of its multitarget effect, traditional Chinese medicine (TCM) has obvious advantages in the treatment of IBS-D. Tongxie Anchang Decoction (TXACD) is a new and effective traditional Chinese medicine (TCM) prescription formulated by Junxiang Li, a professor from Dongfang Hospital, Beijing University of Chinese Medicine. A single-center, randomized positive-controlled clinical trial has shown that TXACD effectively alleviates abdominal pain [16]. However, the exact mechanism of action is yet to be elucidated. Due to the close relationship between the NGF/TrkA signaling pathway and visceral hypersensitivity, we hypothesize that TXACD plays its therapeutic role by regulating the NGF/TrkA signaling pathway. Thus, the present study sought to further explore the molecular mechanism underlying TXACD's protective effects in rat models of IBS-D.

## 2. Materials and Methods

### 2.1. Preparation of TXACD and Pinaverium Bromide

TXACD granules were purchased from the Pharmacy Department of Dongfang Hospital, Beijing University of Chinese Medicine (Beijing, China). The TXACD granules contained Tongxie Anchang ingredients in equal weights: Baizhu (*Atractylodes macrocephala* Koidz), 15 g; Baishao (Paeoniae Radix Alba), 12 g; Huanglian (Coptidis Rhizoma), 6 g; Paojiang (Rhizoma Zingiberis Preparata), 9 g; Chenpi (*Citrus reticulata*), 6 g; Chantui (Cicadae Periostracum), 6 g; and Wumei (Mume Fructus), 9 g. Drug quality control is available. Pinaverium bromide was purchased from Beijing Wansheng Pharmaceutical Co., Ltd. (Beijing, China).

### 2.2. Experimental Animals

Male Sprague–Dawley rats (postnatal day 2) were purchased from SPF Biological Technology Co., Ltd. (Beijing, China). All rats were housed in a specific pathogen-free animal room with temperature maintained at 20–24°C, 50–60% humidity, and a light-controlled environment (12/12 h light/dark cycle), with free access to food and sterile tap water. In the present study, all manipulations were performed between 8 : 00 and 11 : 00 am every day to minimize the effect of circadian rhythm. On postnatal day 22, all the litters were weaned and placed in separate cages with 4-5 rats per cage. All experimental procedures were performed in accordance with the recommendations of the National Institutes of Health guidelines on the care and use of laboratory animals and approved by the Experimental Animal Ethics Committee of Beijing University of Chinese Medicine (BUCM-4-2019092403-309).

### 2.3. Induction of Diarrhea-Predominant Irritable Bowel Syndrome in Rats

The IBS-D model was induced as follows [17, 18]. On postnatal days 4–21, the neonatal maternal separation (NMS) was conducted. The NMS litters were removed from home cages and separated from their maternal rats for 3 hours a day. During the separation, the litters were placed in individual cages. Then, litters returned to home cages after separation. The litters in the normal control group (none handled, NH) stayed in home cages with their maternal rats. And all the NMS rats were treated with restraint stress (RS) on postnatal days 50–59. The restraint stress was performed as follows: the NMS-treated rats were placed in the binding plate for 3 hours and could not move.

### 2.4. Experimental Design

The normal control group (no handling) rats were classified as the NH group. The NMS and RS-treated rats were randomly divided into five groups: NMS + RS group (NR, *n* = 6), pinaverium bromide (PB, 20 mg/kg/day, *n* = 6), low-dose TXACD (low, 3.31 g/kg/day, *n* = 6), middle-dose TXACD (middle, 6.62 g/kg/day, *n* = 6), and high-dose TXACD (high, 13.24 g/kg/day, *n* = 6). The dose was given according to the equivalent dose ratio of human to animal surface area: rat dose = *X* mg/kg × 70 kg × 0.018 kg (*X* was regarded as the adult clinical dose). After postnatal days 60, the TXACD groups and pinaverium bromide were given intragastric administration, respectively, while the NH group and NR group were given distilled water. All groups were treated continuously for 14 days. Then, fecal water content and abdominal withdrawal reflex (AWR) were detected. Subsequently, the rats were sacrificed, and colonic tissues and blood samples were collected for further analysis.

### 2.5. Fecal Indexes

The rats' feces in all 6 groups were collected 24 hours after intragastric administration for 14 days. The collected feces (wet) are weighed immediately and weighed again after drying at 80°C for 10 hours (dry). The formula for calculating fecal water content is as follows: fecal water content = ((wet feces−dry feces)/wet feces) × 100% [18].

### 2.6. Measurement of Visceral Hypersensitivity

After 14 days of treatment, visceral hypersensitivity was evaluated by the abdominal withdrawal reflex (AWR) score system. After deep inhalation anesthesia with isoflurane, an elastic balloon with a paired tube was inserted from the anus to the descending colon, and the tube was affixed to the tail and fixed in place. Rats were placed in a cage and allowed to adapt for 30 minutes. Colorectal distension (CRD) was simulated by rapidly inflating the balloon to constant pressure. The balloon pressure increased successively, the duration of 20, 40, 60, and 80 mmHg was 30 seconds, and the interval between distensions was 180 seconds. The rats' behavioral response to CRD was observed and evaluated by two researchers who were blind to the experimental procedures. AWR scores were measured as follows: 1, normal behavior without response; 2, abdominal muscle contraction; 3, abdominal wall elevation; 4, body arch and pelvic structure elevation.

### 2.7. Western Blotting for Detection of NGF, TrkA, and TRPV1 Expression in Colon

The colonic tissue was extracted with precooled RIPA protein extraction reagent and protease inhibitor; then, the total protein was extracted, and the protein concentration was determined by the bicinchoninic acid (BCA) method. The protein sample was added into 5 × reduction sample buffer and boiled 5 minutes to denature. The proteins were then separated using SDS-PAGE before being transferred to nitrocellulose (NC) membranes. The membranes were probed with the NGF (1 : 1000), TrkA (1 : 1000), TRPV1 (1 : 2000) antibodies (Abcam, Cambridge, UK), and *β*-actin (1 : 5000) antibody (Santa Cruz Biotechnology, California, USA). Each membrane was washed 5 times, 3 minutes each time and incubated with goat polyclonal secondary antibody to rabbit antibodies. Finally, densitometry was performed to quantitate protein band intensities by using the Quantity One image analysis software ver. 4.4 (BioRad Laboratories, California, USA).

### 2.8. Real-Time Polymerase Chain Reaction (PCR) for Detection of NGF, TrkA, and TRPV1 mRNA Expression in Colon

Total RNA was extracted from colon tissue samples by the TRIzol extraction kit. After reverse transcription, PCR amplification was carried out with the real-time PCR system: cDNA 1ul, 2 × mix 12.5 *μ*l, upstream and downstream primers 0.5 *μ*l, respectively, and ddH_2_O 10.5 *μ*l. The real-time primer sequences of the target genes are given in Table 1. For real-time PCR, the cycling conditions were 72°C for 10 minutes, 35 × (94°C for 30 s, 60°C/62°C for 30 s, and 72°C for 30 s). Then, the melting curve was analyzed. The relative expression was assessed by calculating the expression relative to that of GAPDH by using the 2^−ΔΔ^Ct method.

### 2.9. Toluidine Blue Staining

The colon specimens fixed in paraformaldehyde were taken, and paraffin-embedded tissue sections were made. After xylene dewaxing and gradient alcohol (100–75%) dewaxing to water, the sections were stained with toluidine blue for 5 minutes. Subsequently, the following procedures were manipulated successively, including glacial acetic acid differentiation, xylene transparent, and neutral resin seal. As a result, MC was stained purplish red and the nucleus was blue. For each section, three random fields (400 × objective magnification) were viewed using a CaseViewer (3DHISTECH Ltd., Version: 2.0, Budapest, Hungary), and the mean value of mast cell counts was determined by two independent and blinded researchers. The results were expressed as the mean number of mast cells/high-power field.

### 2.10. Measurement of Serum SP, 5-HT, and CGRP Levels Using ELISA

After blood samples were collected from the abdominal aorta of rats; the serum SP, 5-HT, and CGRP levels were tested using enzyme-linked immunosorbent assay (ELISA) kits (Shanghai Enzyme-linked Biotechnology, Shanghai, China).

### 2.11. Statistical Analysis

The data were analyzed using IBM SPSS Statistics (Version 24.0 position IBM Corp., Armonk, NY), and the data were expressed as mean ± standard deviation (SD) values. One-way analysis of variance (ANOVA) was used for comparison between groups. *P* < 0.05 was considered statistically significant.

## 3. Results

### 3.1. Effect of TXACD on Fecal Indexes

To confirm successful induction of the IBS-D model in rats and evaluate the therapeutic effect of TXACD, the wet feces weight and fecal water content of experimental IBS-D rats were measured. As given in Table 2, the wet feces weight in the NR group was significantly increased from 8.74 ± 0.33 to 11.59 ± 0.59 g (*P* < 0.01), and fecal water content increased from 16.15 ± 1.09% to 33.26 ± 0.81% (*P* < 0.01), comparing to the NH group, indicating that IBS-D rats were induced successfully. After treatment, the wet feces weight and fecal water content in the TXACD group and PB group were significantly decreased than those in the NR group (*P* < 0.01). These results suggested that the symptoms of the IBS-D model improved effectively by TXACD.

### 3.2. Effect of TXACD on Visceral Hypersensitivity

The visceral hypersensitivity was measured with the AWR score system based on the behavioral response of rats to CRD. As given in Table 3, there was no significant difference in AWR scores among the three groups under the distention pressure of 20 mmHg. However, the AWR scores in the NR group were significantly higher than that in the NH group when the distention pressure was 40, 60, and 80 mmHg (*P* < 0.01). After TXACD intervention, compared with the NR group, the AWR scores decreased significantly under the distension pressure of 40, 60, and 80 mmHg (*P* *<* 0.01), except for the low-dose TXACD (*P* *>* 0.05). These results indicated that TXACD significantly improve visceral hypersensitivity.

### 3.3. Effect of TXACD on the NGF/TrkA Signaling Pathway

As mentioned previously, the NGF/TrkA signaling pathway is crucial for the formation of visceral hypersensitivity. To elucidate whether the role of TXACD was associated to with the NGF/TrkA signaling pathway, we measured the protein and gene expression levels of NGF and TrkA.

#### 3.3.1. Effect of TXACD on the Expression and Function of NGF in Colon

**Figure 1:**
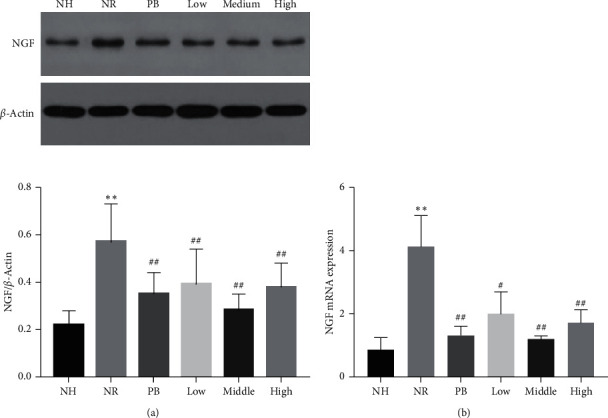
Effects of TXACD on NGF protein and mRNA levels in rats. (a) The protein levels of NGF were determined by Western blotting analysis. (b) The mRNA levels of the NGF were determined by real-time PCR. ^*∗∗*^*P* < 0.01 versus the normal control (no handling (NH)) group. ^#^*P* < 0.05 and ^##^*P* < 0.01 versus the neonatal maternal separation (NMS) + restraint stress (RS) (NR) group.

As shown in Figure 1, compared with the NH group, the protein and gene expression levels of the NGF in the NR group were significantly increased (*P* < 0.01). However, TXACD treatment significantly reversed this increase (*P* < 0.01 and *P* < 0.05).

#### 3.3.2. Effect of TXACD on the Expression and Function of TrkA in Colon

**Figure 2:**
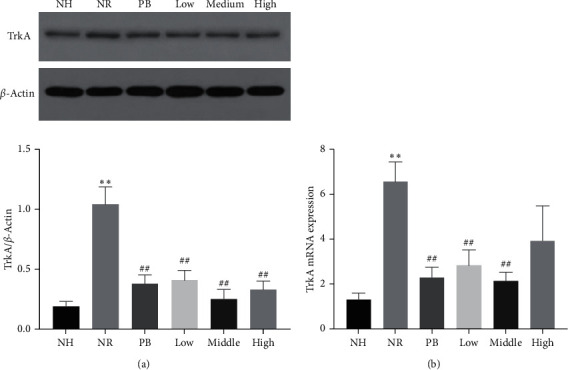
Effects of TXACD on TrkA protein and mRNA levels in rats. (a) The protein levels of TrkA were determined by Western blotting analysis. (b) The mRNA levels of TrkA were determined by real-time PCR. ^*∗∗*^*P* < 0.01 versus the normal control (no handling (NH)) group. ^##^*P* < 0.01 versus the neonatal maternal separation (NMS) + restraint stress (RS) (NR) group.

As shown in Figure 2(a), the protein expression level of TrkA in the NR group was significantly increased, compared with the NH group (*P* < 0.01). After treatment, TXACD significantly reversed this situation (*P* < 0.01). As shown in Figure 2(b), compared with the NH group, the gene expression level of TrkA was significantly increased in the NR group (*P* < 0.01). Following TXACD treatment, low-dose and middle-dose TXACD significantly reversed this increase (*P* < 0.01). These results indicated that the therapeutic effect of TXACD was associated with the expression and function of the NGF and TrkA.

### 3.4. Effect of TXACD on the Expression and Function of TRPV1 in Colon

**Figure 3:**
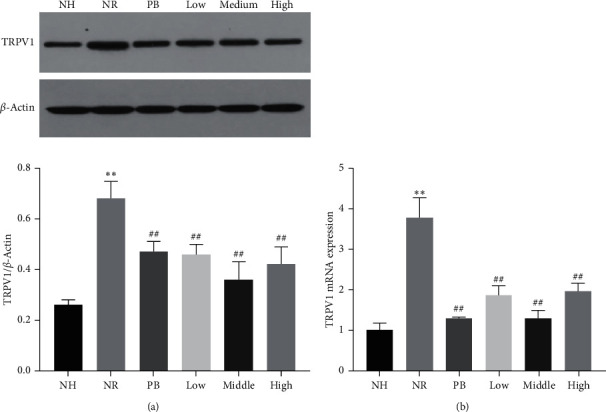
Effects of TXACD on TRPV1 protein and mRNA levels in rats. (a) The protein levels of TRPV1 were determined by Western blotting analysis. (b) The mRNA levels of TRPV1 were determined by real-time PCR. ^*∗∗*^*P* < 0.01 versus the normal control (no handling (NH)) group. ^##^*P*< 0.01 versus the neonatal maternal separation (NMS) + restraint stress (RS) (NR) group.

The degree of abdominal pain in patients with IBS-D was positively correlated with the expression of TRPV1 in colon, and the activation of TRPV1 led to the influx of Ca^2+^, which was directly involved in the occurrence of visceral hypersensitivity. To determine whether the expression and function of TRPV1 was affected by TXACD, the protein and gene expression levels of TRPV1 were detected by Western blotting and real-time PCR analyses. As shown in Figure 3, compared with the NH group, the protein and gene expression levels of TRPV1 in the NR group were significantly increased (*P* < 0.01). However, TXACD treatment significantly reversed this increase (*P* < 0.01). These results showed that TXACD played a therapeutic role by regulating the expression of TRPV1.

### 3.5. Effect of TXACD on Mast Cell Infiltration in Colon

**Figure 4:**
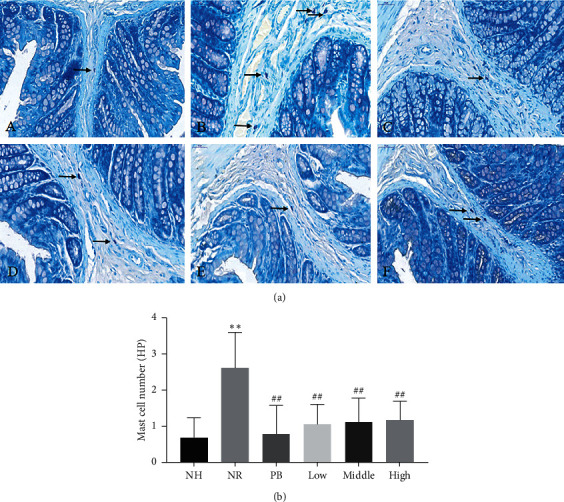
(a) Toluidine blue staining for mast cells. A, NH group; B, NR group; C, PB group; D, low-dose TXACD group; E, middle-dose TXACD group; F, high-dose TXACD group. (b) Effect of TXACD on mast cells infiltration in colon. ^*∗∗*^*P* < 0.01 versus the normal control (no handling (NH)) group. ^##^*P* < 0.01 versus the neonatal maternal separation (NMS) + restraint stress (RS) (NR) group.

Mucosal mast cells (MC) play a crucial part in the development of heightened visceral sensitivity. Compared with the NH group, MC counts in the NR group were significantly increased (2.61 ± 0.98/HP in the NR group versus 0.67 ± 0.56/HP). As shown in Figure 4, after TXACD treatment, mast cells infiltration was significantly improved.

### 3.6. Effect of TXACD on the Serum Levels of 5-HT, CGRP, and SP

**Figure 5:**
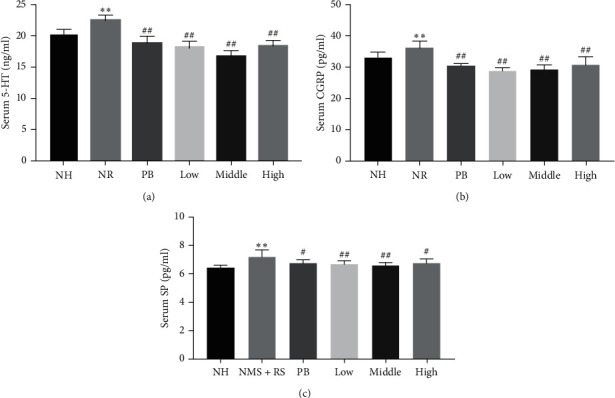
Effects of TXACD on serum 5-HT, CGRP, and SP levels in rats. The serum 5-HT, CGRP, and SP levels were determined by ELISA. (a) Serum 5-HT level. (b) Serum CGRP level. (c) Serum SP level. ^*∗∗*^*P* < 0.01 versus the normal control (no handling (NH)) group. ^##^*P* < 0.01 versus the neonatal maternal separation (NMS) + restraint stress (RS) (NR) group.

It was reported that neuroendocrine mediators (such as 5-HT, CGRP, and SP) played a key role in activating intestinal sensory nerve endings and participating in the perception of intestinal visceral pain. We detected the serum levels of 5-HT, CGRP, and SP by ELISA to determine whether the expression of neuroendocrine mediators was affected by TXACD. As shown in Figure 5(a), the serum 5-HT level in the NR group was significantly increased than that in the NH group (22.46 ± 0.79 ng/ml in the NR group versus 20.16 ± 0.84 ng/ml). The low-dose TXACD group (18.11 ± 1.02 ng/mL), the middle-dose TXACD group (16.69 ± 0.94 ng/mL), and the high-dose QCWZD group (18.39 ± 0.85 ng/mL) showed significant inhibition of serum 5-HT level elevation (versus the NR group, *P* < 0.01). As shown in Figures 5(b) and 5(c), compared with the NH group, NR-induced rats significantly increased the serum CGRP and SP levels (*P* < 0.01). Following TXACD treatment, the increase of serum CGRP and SP levels were reversed. Collectively, these results indicated that the therapeutic effect of TXACD was associated with the reduction of serum 5-HT, CGRP, and SP levels.

## 4. Discussion

In the present study, we found that TXACD had a therapeutic effect on IBS-D rats. Physiologically, TXACD alleviated visceral hypersensitivity in IBS-D rats. Mechanistically, TXACD modulated visceral hypersensitivity by controlling the release of neuroendocrine mediators through the NGF/TrkA signaling pathway. In clinical research, we found that TXACD effectively relieved abdominal pain and other symptoms in patients with IBS-D [16]. In the experimental study, we also observed that TXACD significantly improved fecal indexes and reduced the AWR score after CRD-treated.

The pathogenesis of IBS-D involves a variety of pathophysiological processes. In recent years, more and more attention has been paid to the mechanism of the neuro-immune-endocrine network, especially the role of various neuroendocrine mediators, such as the nerve growth factor (NGF), 5-HT, SP, and CGRP. Some studies [19, 20] have shown that mediators such as the NGF and 5-HT have the function of activating intestinal sensory nerve endings. Moreover, as a member of the neurotrophic factors family, the NGF is able to change the plasticity of local neurons and affect the distribution of sensory nerve endings, the expression of nociceptive receptor proteins (such as TRPV1), and the release of neuropeptides (such as CGRP and SP) [21]. These pathological factors lead to the hypersensitivity of sensory nerve endings to nociceptive and even physiological stimuli, which form the basis of visceral hypersensitivity. Neonatal maternal separation (NMS) of rodents is a common animal model for the study of stress-related diseases in early life, which lead to a variety of gastrointestinal dysfunction, including visceral hypersensitivity to CRD and increased colonic motility [17, 22]. Stress animal models present similar pathophysiologic abnormalities to that in IBS-D patients. Therefore, we used NMS + RS-treated rats to establish the IBS-D rat model [18, 23].

The NGF belongs to the family of neurotrophic factors, which is composed of *α*, *β*,  and *δ* subunits and is distributed in the brain, ganglion, intestine, and other organs. NGF binding to the receptor TrkA produces biological functions. Dothel et al. [24] found that mucosal NGF and TrkA levels significantly increased in patients with IBS compared to the control group. In addition, under acute or chronic stress and other pathological conditions, increased the NGF acted on the intestinal sensory nerve endings, promoted their growth and synapse formation, and thus mediated visceral hypersensitivity [25]. Barreau et al. [26] reported that the NGF played an crucial role in the formation of visceral hypersensitivity induced by NMS. After several weeks of NMS-induced visceral hypersensitivity and colonic NGF increased, and anti-NGF antibody treatment during NMS could eliminate NMS-induced hypersensitivity. In the present study, compared with the NH group, the protein and gene expression level of the NGF and TrkA in the NR group increased significantly, which was consistent with the results of clinical studies. What is more, after treatment, TXACD could reverse this increase. These results demonstrated that the therapeutic effect of TXACD was closely related with the expression and function of the NGF and TrkA.

TRPV1 (transient receptor potential vanilloid 1) belongs to the family of transient receptor potential channels (transient receptor potential, TRP), which is mainly stimulated by chemical injury. Akbar et al. [27] reported that SP-positive nerve fibers and TRPV1-positive nerve fibers increased in colonic mucosa of IBS patients. What is more, the degree of abdominal pain was positively correlated with the intensity of TRPV1 staining, speculating that the NGF mediated upregulation of TRPV1 expression in low-grade inflammatory mucosa of IBS, resulting in visceral hypersensitivity. Other studies found that the activation of TRPV1 led to the influx of Ca2^+^, the local release of CGRP and SP, and the activation of effector cell receptors such as mast cells (MC) in colon. MC played a crucial part in signal transduction of the gut-brain axis. What is more, because of their proximity to the nerve, MC might affect the smooth muscle and enteric nerves by releasing potent mediators. It was reported that MC were closely associated with the induced changes in nerve function and the development of heightened visceral sensitivity [10]. In a word, the expression and function of TRPV1 and MC infiltration played an important part in the pathological processes of neurogenic inflammation and visceral sensitivity [28, 29]. Our study demonstrated that the protein and gene expression level of TRPV1 in the NR group was significantly increased than that in the NH group. Following TXACD treatment, the expression level of TRPV1 significantly decreased. MC number in the NR group was significantly higher than that in the NH group. After TXACD treatment, mast cells infiltration was significantly improved. In addition, compared to the NH group, the serum SP and CGRP levels in the NR group were significantly elevated. However, TXACD treatment showed significant inhibition of NR-induced elevation of serum SP and CGRP levels. These results suggested that TXACD played a therapeutic role in alleviating visceral hypersensitivity by downregulating the expression of TRPV1, improving mast cells infiltration in colon, and reducing the release of CGRP and SP.

In the present study, the serum 5-HT level was significantly increased in NR-induced rats, which was consistent with the literature report. Moreover, the serum 5-HT level elevation was inhibited after TXACD treatment. These results indicated that TXACD improved visceral hypersensitivity by reducing the release of 5-HT. 5-HT has been shown to be closely related to the pathogenesis of IBS by affecting intestinal motility and nociceptive sensation. Chow et al. [30] found that upregulation of the NGF/TrkA signaling pathway caused by early life stress (such as NMS) promoted the differentiation of intestinal stem cell (ISCs) into intestinal chromaffin cells (enterochromaffin cells, EC). Increased 5-HT, produced by the enlarged EC cells population, led to hyperalgesia and enhanced colonic motility [31], so the change of 5-HT signal resulted in the visceral hypersensitivity of IBS-D [32].

## 5. Conclusions

To sum up, the present study demonstrated that TXACD was an effective agent for ameliorating visceral hypersensitivity in IBS-D rats. The protective mechanism might be associated to the downregulation of the NGF/TrkA signaling pathway, reversal of TRPV1 expression and mast cells infiltration, and decreased release of neuroendocrine factors 5-HT, SP, and CGRP.

## Figures and Tables

**Table 1:** List of primers for real-time PCR.

Gene name	Forward primer (5′–3′)	Reverse primer (5′–3′)
NGF	GGCATTGACTCCAAGCAC	GTATCTATCCTGATGAACCTCC
TrkA	TGGCAGCGTGAGCCGCAACA	GCCCAGAACGTCCAGGTAA
TRPV1	GTGGAACCCTTGAACCGACT	AACTCTTGAGGGATGGTCGC
GAPDH	ACAGCAACAGGGTGGTGGAC	TTTGAGGGTGCAGCGAACTT

**Table 2:** Effect of TXACD on fecal indexes of rats.

Group	*n*	Wet feces weight (g)	Fecal water content (%)
NH	6	8.74 ± 0.33	16.15 ± 1.09
NR	6	11.59 ± 0.59^*∗∗*^	33.26 ± 0.81^*∗∗*^
Low dose	6	9.48 ± 0.23^##^	24.67 ± 0.79^##^
Middle dose	6	8.94 ± 0.29^##^	22.04 ± 0.80^##^
High dose	6	9.15 ± 0.38^##^	23.87 ± 1.22^##^
Pinaverium bromide (PB)	6	8.77 ± 0.30^##^	22.37 ± 1.03^##^

^*∗∗*^ *P* < 0.01 versus the normal control (no handling (NH)) group. ^##^*P* < 0.01 versus the neonatal maternal separation (NMS) + restraint stress (RS) (NR) group.

**Table 3:** Effect of TXACD on AWR score in rats.

Group	*n*	20 mmHg	40 mmHg	60 mmHg	80 mmHg
NH	6	0.56 ± 0.27	1.39 ± 0.25	1.89 ± 0.40	2.56 ± 0.46
NR	6	1.22 ± 0.34	2.56 ± 0.34^*∗∗*^	3.39 ± 0.39^*∗∗*^	3.89 ± 0.40^*∗∗*^
Low-dose	6	1.11 ± 0.40	2.22 ± 0.54	3.28 ± 0.25	3.83 ± 0.62
Middle dose	6	1.06 ± 0.44	1.67 ± 0.30^##^	2.28 ± 0.25^##^	2.94 ± 0.49^##^
High dose	6	1.11 ± 0.40	1.67 ± 0.37^##^	2.72 ± 0.39^##^	3.17 ± 0.46^##^
Pinaverium bromide (PB)	6	1.06 ± 0.39	1.61 ± 0.25^##^	2.06 ± 0.44^##^	2.72 ± 0.39^##^

^*∗∗*^ *P* < 0.01 versus the normal control (no handling (NH)) group. ^##^*P* < 0.01 versus the neonatal maternal separation (NMS) + restraint stress (RS) (NR) group.

## Data Availability

The datasets used and/or analyzed during the current study are available from the corresponding author upon request.

## Supplementary Materials

Supplementary Materials Figure NGF 1 is chosen as the manuscript image. Figures NGF 1–6 represent all the original Western blot figures of NGF. Figure TrkA 1 is chosen as the manuscript image. Figures TrkA 1–6 represent all the original Western blot figures of TrkA. Figure TRPV1 4 is chosen as the manuscript image. Figures TRPV1 1–6 represent all the original Western blot figures of TRPV1. NGF and TrkA share the same one *β*-actin. Figure *β*-actin 1 is chosen as the manuscript image of *β*-actin of NGF and TrkA. Figures *β*-actin 1–6 represent all the original Western blot figures of *β*-actin of NGF and TrkA. For the two blots in figures, *β*-actin 2, *β*-actin 3, *β*-actin 5, and *β*-actin 6, the upper blot represents the *β*-actin of NGF and TrkA. The blot in the left represents the *β*-actin of NGF and TrkA. Figure *β*-actin 4 is chosen as the manuscript image of *β*-actin of TRPV1. Figures *β*-actin 1–6 represent all the original Western blot figures of *β*-actin of TRPV1. For the two blots in figures, *β*-actin 2, *β*-actin 3, *β*-actin 5, and *β*-actin 6, the lower blot represents the *β*-actin of TRPV1. The blot in the right represents the *β*-actin of TRPV1.
