# Supplementary material for: Tongxie Anchang Decoction Relieves Visceral Hypersensitivity in Diarrhea-Predominant Irritable Bowel Syndrome Rats by Regulating the NGF/TrkA Signaling Pathway
Source: Evid Based Complement Alternat Med. 2021 Jun 18;2021:6679348. doi: 10.1155/2021/6679348 (PMC8235976; doi:10.1155/2021/6679348)
Supplement: Supplementary Materials — Figure NGF 1 is chosen as the manuscript image. Figures NGF 1–6 represent all the original Western blot figures of NGF. Figure TrkA 1 is chosen as the manuscript image. Figures TrkA 1–6 represent all the original Western blot figures of TrkA. Figure TRPV1 4 is chosen as the manuscript image. Figures TRPV1 1–6 represent all the original Western blot figures of TRPV1. NGF and TrkA share the same one β-actin. Figure β-actin 1 is chosen as the manuscript image of β-actin of NGF and TrkA. Figures β-actin 1–6 represent all the original Western blot figures of β-actin of NGF and TrkA. For the two blots in figures, β-actin 2, β-actin 3, β-actin 5, and β-actin 6, the upper blot represents the β-actin of NGF and TrkA. The blot in the left represents the β-actin of NGF and TrkA. Figure β-actin 4 is chosen as the manuscript image of β-actin of TRPV1. Figures β-actin 1–6 represent all the original Western blot figures of β-actin of TRPV1. For the two blots in figures, β-actin 2, β-actin 3, β-actin 5, and β-actin 6, the lower blot represents the β-actin of TRPV1. The blot in the right represents the β-actin of TRPV1. [file 6679348.f1.pptx]

## Slide 1
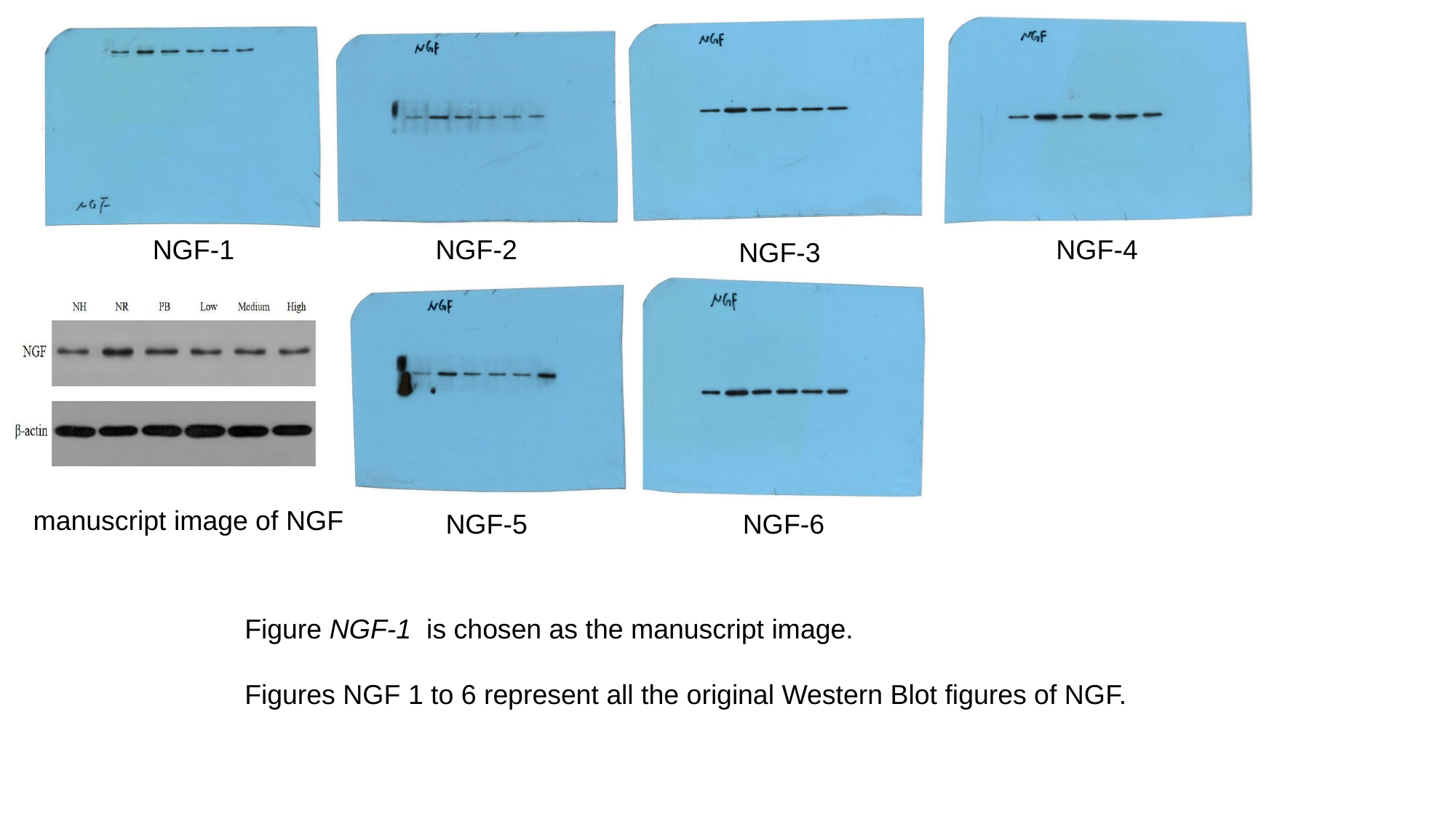

NGF-1
NGF-2
NGF-4
NGF-3
manuscript image of NGF
NGF-5
NGF-6
Figure NGF-1 is chosen as the manuscript image.
Figures NGF 1 to 6 represent all the original Western Blot figures of NGF.

## Slide 2
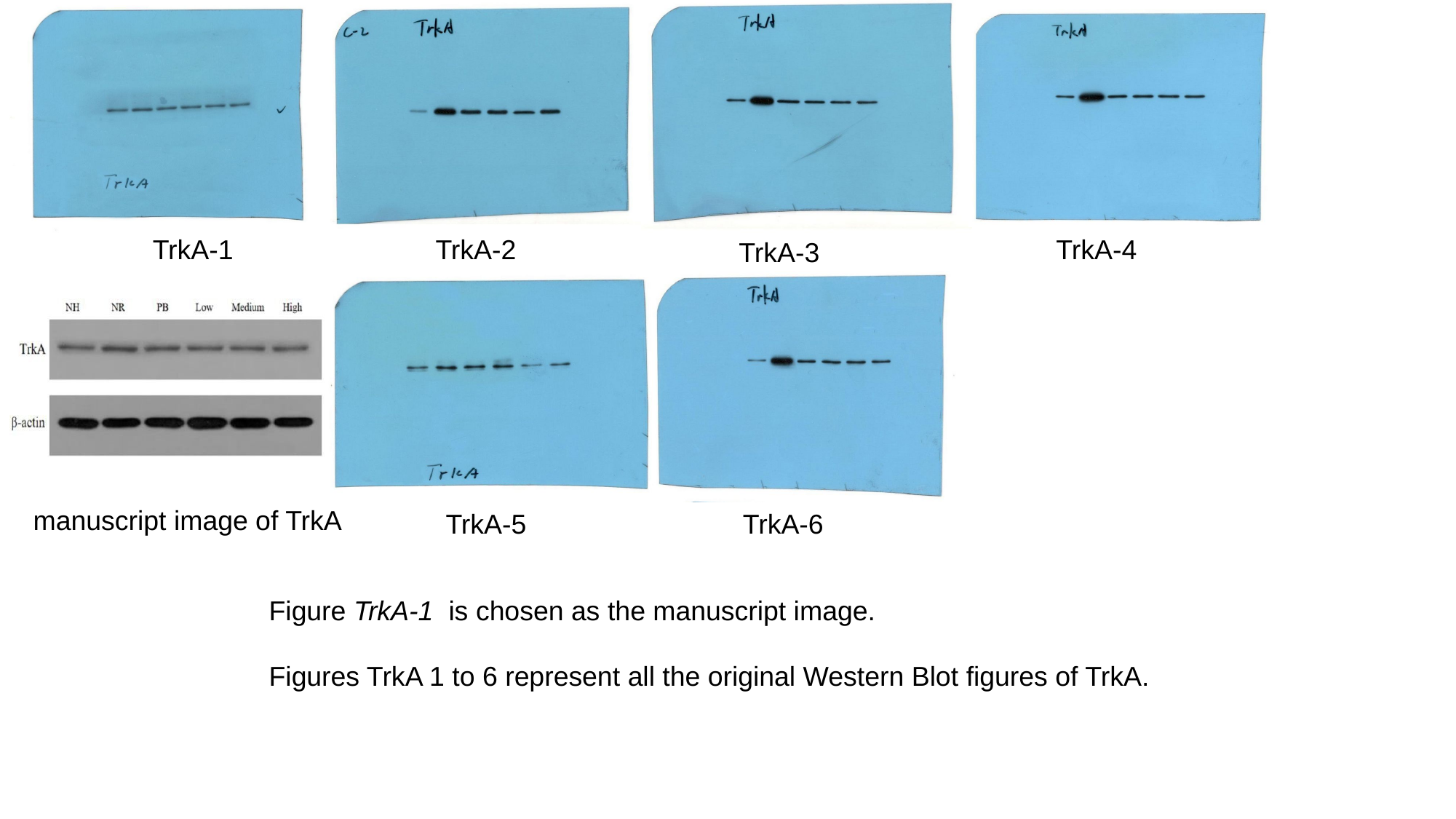

TrkA-1
TrkA-2
TrkA-4
TrkA-3
manuscript image of TrkA
TrkA-5
TrkA-6
Figure TrkA-1 is chosen as the manuscript image.
Figures TrkA 1 to 6 represent all the original Western Blot figures of TrkA.

## Slide 3
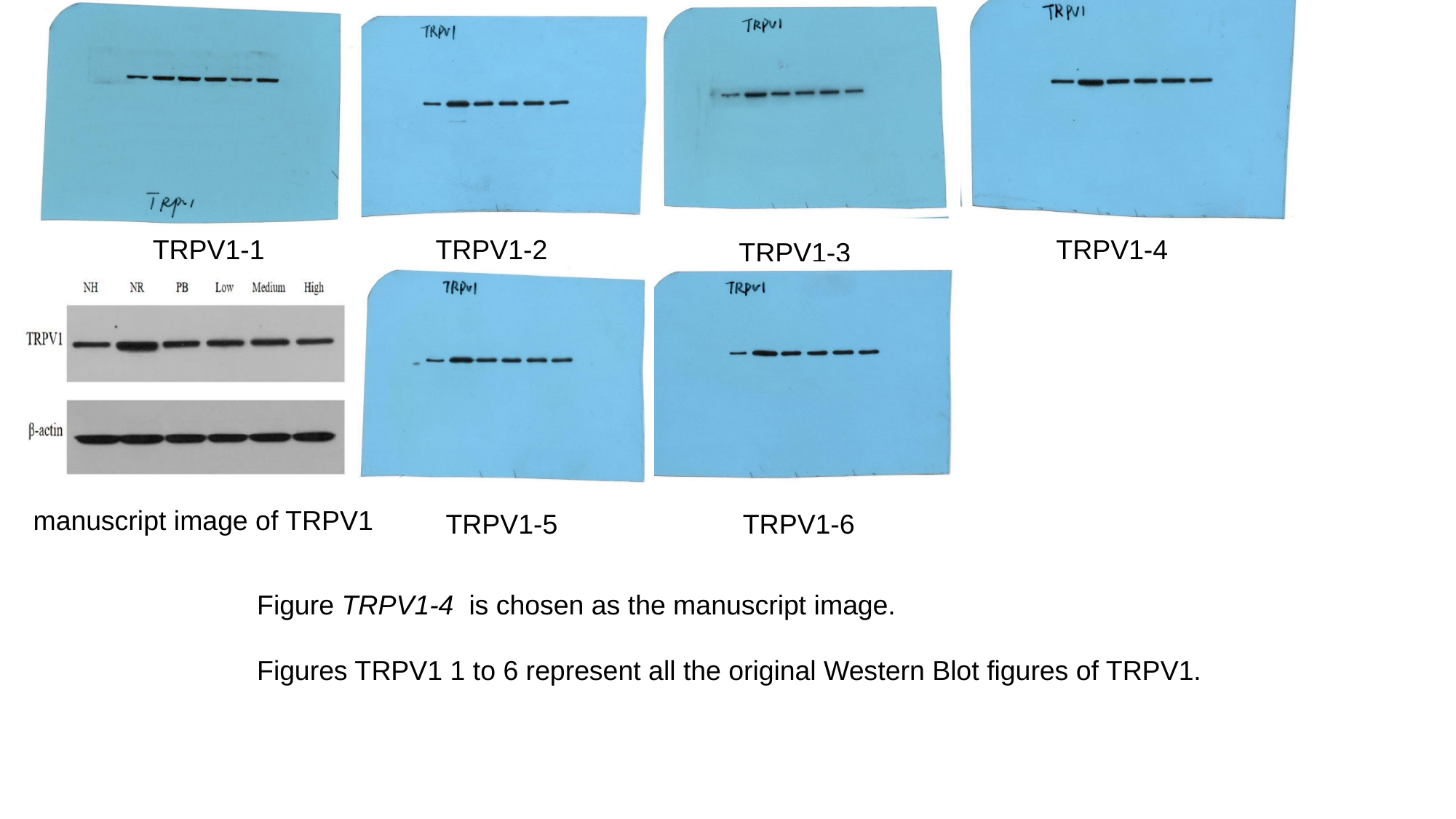

TRPV1-1
TRPV1-2
TRPV1-4
TRPV1-3
manuscript image of TRPV1
TRPV1-5
TRPV1-6
Figure TRPV1-4 is chosen as the manuscript image.
Figures TRPV1 1 to 6 represent all the original Western Blot figures of TRPV1.

## Slide 4
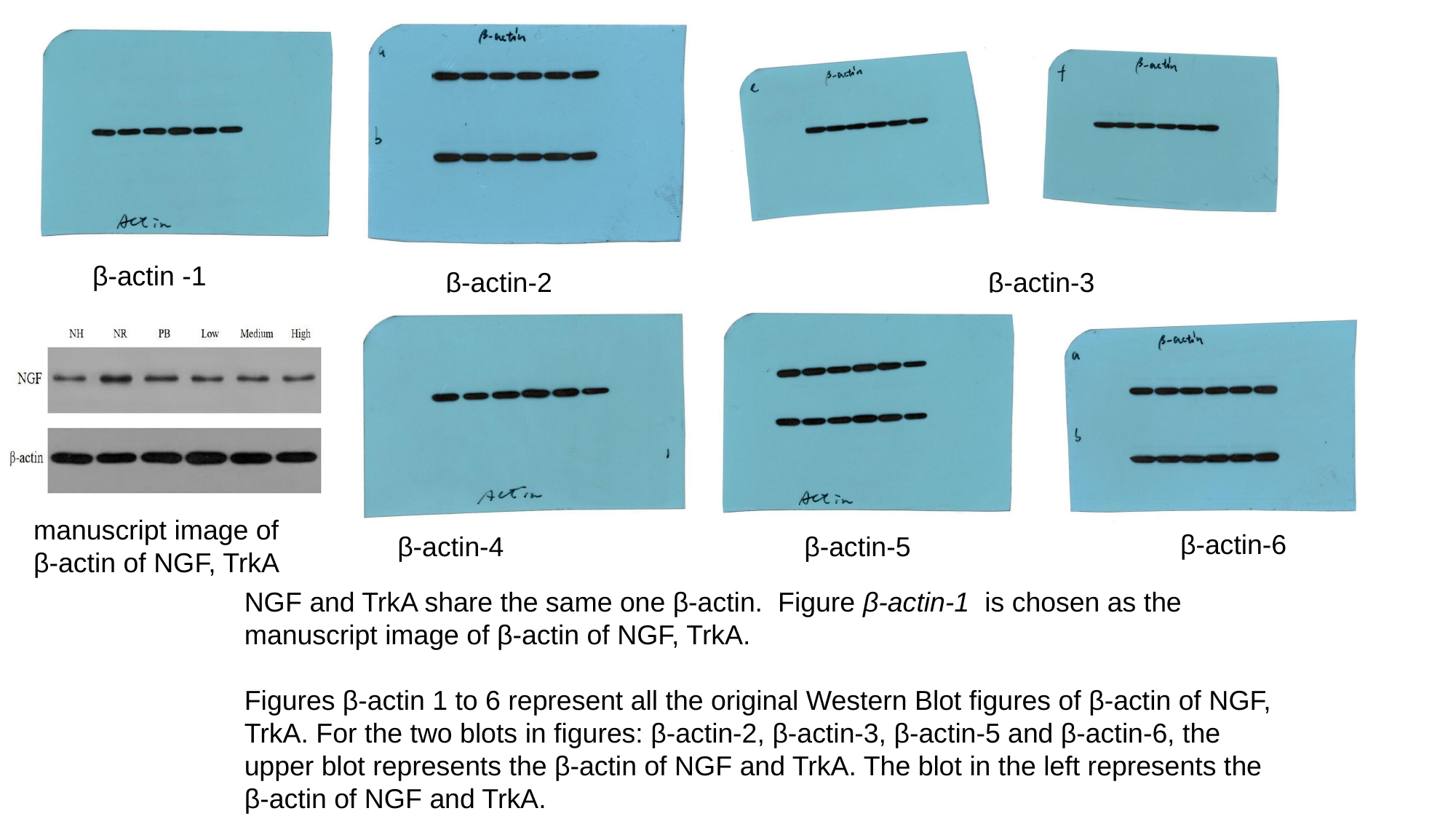

β-actin -1
β-actin-2
β-actin-3
manuscript image of
β-actin of NGF, TrkA
β-actin-6
β-actin-4
β-actin-5
NGF and TrkA share the same one β-actin. Figure β-actin-1 is chosen as the manuscript image of β-actin of NGF, TrkA.
Figures β-actin 1 to 6 represent all the original Western Blot figures of β-actin of NGF, TrkA. For the two blots in figures: β-actin-2, β-actin-3, β-actin-5 and β-actin-6, the upper blot represents the β-actin of NGF and TrkA. The blot in the left represents the β-actin of NGF and TrkA.

## Slide 5
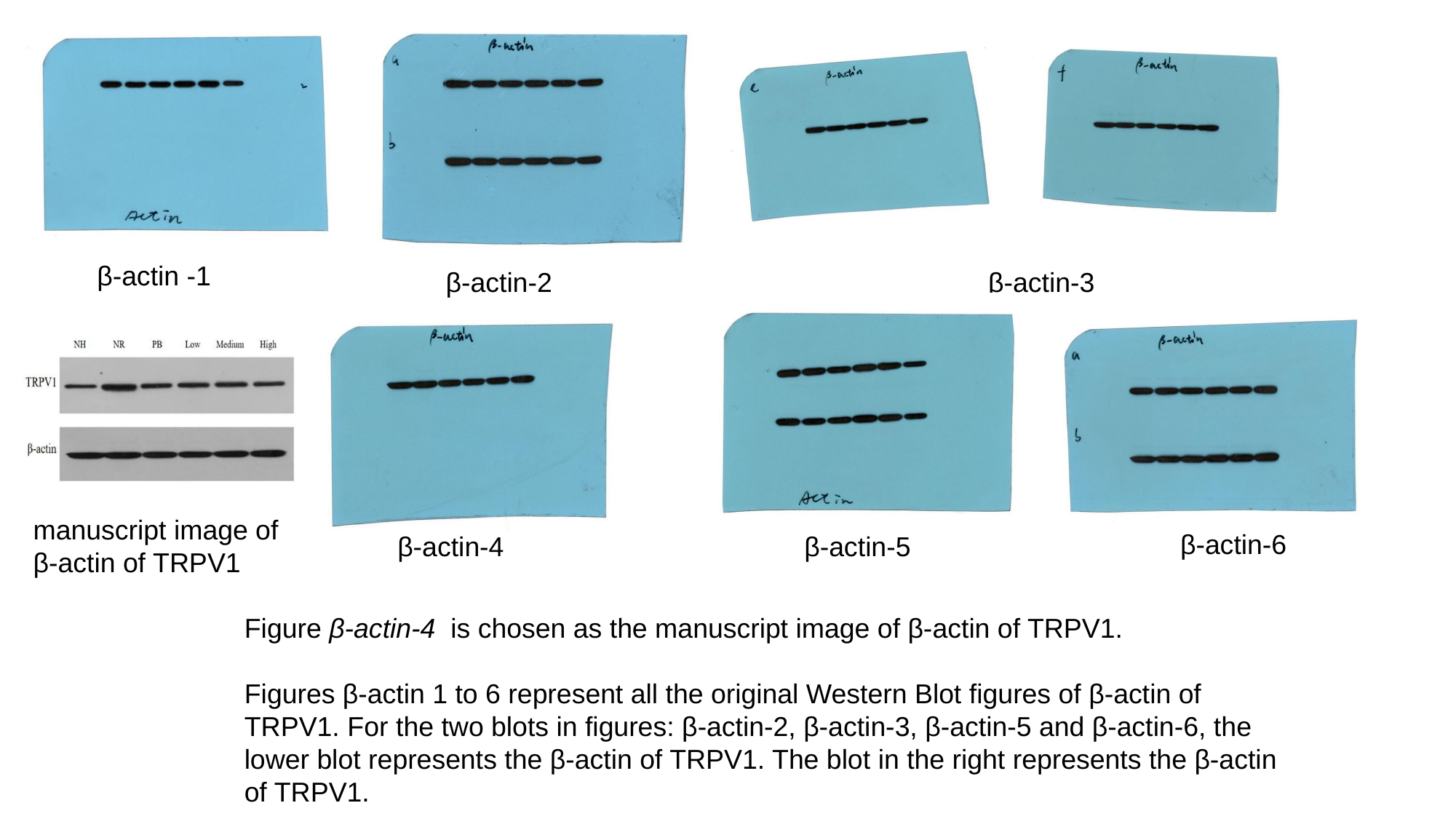

β-actin -1
β-actin-2
β-actin-3
manuscript image of
β-actin of TRPV1
β-actin-6
β-actin-4
β-actin-5
Figure β-actin-4 is chosen as the manuscript image of β-actin of TRPV1.
Figures β-actin 1 to 6 represent all the original Western Blot figures of β-actin of TRPV1. For the two blots in figures: β-actin-2, β-actin-3, β-actin-5 and β-actin-6, the lower blot represents the β-actin of TRPV1. The blot in the right represents the β-actin of TRPV1.
